# A Serum N‐Glycan Ratio Associated With Disease Severity and Treatment Response in Psoriasis

**DOI:** 10.1111/1346-8138.70316

**Published:** 2026-06-23

**Authors:** Kentarou Nishimura, Noritaka Oyama, Takumi Hasegawa, Hiroshi Kasamatsu, Takenao Chino, Takahiro Tokunaga, Kiyoshi Higashi, Koichi Saito, Keita Yamada, Minoru Hasegawa

**Affiliations:** ^1^ Department of Dermatology, Division of Medicine, Faculty of Medical Sciences University of Fukui Fukui Japan; ^2^ Research Promotion Office Shinseikai Toyama Hospital Toyama Japan; ^3^ Medical Research Support Center University of Fukui Hospital Fukui Japan; ^4^ Advanced Materials Development Lab Sumitomo Chemical Co., Ltd Osaka Japan; ^5^ Laboratory of Toxicology Osaka Ohtani University Osaka Japan

**Keywords:** biomarkers, CCL20, CRP, N‐linked glycans, psoriasis

## Abstract

Psoriasis is a chronic, immune‐mediated inflammatory disease with heterogeneous manifestations. Reliable biomarkers reflecting disease severity and treatment response remain limited. Glycans—carbohydrate structures attached to proteins and lipids—play key roles in biological processes, contributing to functional specificity. Abnormal glycosylation has been implicated in the pathogenesis of inflammatory and neoplastic diseases, highlighting their potential as therapeutic targets and biomarkers. This study aimed to evaluate serum N‐glycan profiles as potential biomarkers for psoriasis, focusing on the ratio of sialylated biantennary N‐glycan (S) to fucosylated asialo‐biantennary N‐glycan (FA), termed the S/FA ratio. Serum samples from 45 patients with psoriasis, 12 with atopic dermatitis (AD), and 19 healthy controls were analyzed using high‐performance liquid chromatography. The performance of the S/FA ratio was compared with reported biomarkers (CRP and CCL20), and its associations with Psoriasis Area and Severity Index (PASI) and treatment response were examined. The S/FA ratio was significantly higher in psoriasis than in healthy controls (*p* < 0.001) and correlated with PASI (*r* = 0.485, *p* < 0.001) and its components. Receiver operating characteristic analysis showed comparable diagnostic accuracy among the S/FA ratio (AUC = 0.788, sensitivity: 73.7%, specificity: 73.3%), CRP (AUC = 0.780), and CCL20 (AUC = 0.741). Changes in the S/FA ratio were correlated with improvements in PASI after systemic treatment (*r* = 0.467, *p* = 0.002), including a patients subgroup receiving IL‐23 inhibitors. The S/FA ratio was not significantly influenced by demographics, comorbidities, or arthralgia. No significant difference was observed between psoriasis and AD, and the S/FA ratio was not significantly elevated in AD compared with healthy controls. The serum S/FA ratio may serve as a glycan‐based biomarker associated with disease severity and treatment response in psoriasis, although its ability to distinguish psoriasis from other inflammatory diseases requires further validation in larger and treatment‐naïve cohorts.

## 1 Introduction

Psoriasis is a chronic immune‐mediated inflammatory disease with diverse cutaneous and articular manifestations [1]. Diagnosis is typically based on characteristic skin lesions and, when necessary, histopathological findings from biopsy. While this approach enables accurate diagnosis in most cases, early‐stage, mild, or atypical presentations can be challenging to diagnose. Moreover, in both clinical practice and clinical trials, disease severity and treatment response are primarily assessed based on clinical assessment. Although molecular‐targeted therapies and biologics have markedly improved outcomes in severe, refractory psoriasis, translating advances in molecular‐targeted therapies into reliable biomarkers that reflect disease severity and treatment response remains difficult.

Numerous studies have identified several peripheral blood biomarkers for psoriasis. C‐reactive protein (CRP) is a representative marker of systemic inflammation [2, 3]. A meta‐analysis demonstrated that circulating IL‐2, IL‐17, IL‐18, and IFN‐γ levels may serve as potential biomarkers of psoriasis among inflammatory cytokines [4]. Previous studies have shown that plasma C‐C motif chemokine ligand 20 (CCL20) levels were elevated and correlated with disease severity in patients with psoriasis [5]. A recent proteomic study analyzing 273 plasma proteins identified CCL20 as strongly associated with psoriasis severity, as measured by the PASI, and showed the closest correlation with vascular inflammation [6].

N‐linked glycans (N‐glycans) are carbohydrate chains attached to asparagine residues in proteins, playing critical roles in protein folding, stability, and secretion. Glycosylation of immunoglobulin G (IgG) modulates its effector functions, thus being involved in the processes linking innate and adaptive immunity [7]. Altered IgG N‐glycosylation patterns have been associated with the diagnostic and prognostic value in autoimmune diseases, infections, and cancer [7, 8]. Since N‐glycans are relatively stable in blood, they are suitable for long‐term storage and large‐scale studies. Furthermore, they can be readily extracted and analyzed, enabling noninvasive testing with minimal patient burden.

Recent evidence suggests that N‐glycan profiling may be useful in psoriasis [9]. Comparative analyses between patients with psoriasis and healthy controls have revealed significant differences in specific N‐glycan structures, suggesting a potential association with disease severity.

In this study, we investigate whether structural alterations in serum N‐glycans are associated with psoriasis and evaluate their potential utility for assessing disease severity and treatment response in comparison with previously reported biomarkers.

## 2 Methods

### 2.1 Preparation of Serum‐Derived N‐Glycans

To 15 μL of serum from each subject, 485 μL of purified water was added and gently mixed on ice. The mixture was transferred to an AMICON ULTRA‐0.5 filter unit (Merck; catalog number: C82301) and centrifuged at 14,000 ***g*** for 25 min. The concentrate was diluted with 420 μL of purified water and centrifuged again for further concentration. The resulting concentrate was then dried under reduced pressure. To each sample, 70 μL of purified water, 8 μL of 10% SDS, and 0.6 μL of 2‐mercaptoethanol were added and mixed on ice, followed by heating at 100°C for 10 min.

Subsequently, 8 μL of 10% Nonidet P‐40 aqueous solution and 10 μL of 1 M phosphate buffer (pH 7.5) were added to the reaction mixture, followed by the addition of 3.5 U of N‐Glycosidase F (Roche; catalog number: 06538355103). The mixture was incubated at 37°C for 12 h. After incubation, the reaction mixture was heated at 100°C for 10 min, then mixed with 232 μL of 100% ethanol and centrifuged at 18,000 ***g*** for 10 min. The supernatant was collected, dried under reduced pressure, and used as the serum‐derived N‐glycan.

### 2.2 Fluorescent Labeling of Serum‐Derived N‐Glycans

To the serum‐derived N‐glycan fraction, 15 μL of purified water and 100 μL of 2‐aminobenzoic acid (2AA) reaction solution (3% 2AA, 4% sodium acetate, 2% boric acid, and 3% sodium cyanoborohydride) were added. The mixture was mixed and incubated at 80°C for 90 min. After incubation, 100 μL of purified water was added. The mixture was then applied to a Sephadex LH‐20 column (0.75 cm diameter, 30 cm length), and the fluorescent fractions were collected and dried under reduced pressure. The obtained fractions were used as serum‐derived 2AA‐labeled N‐glycans.

### 2.3 Glycomic High‐Performance Liquid Chromatography (HPLC) Assay

Serum‐derived 2AA‐labeled N‐glycans were analyzed using an Asahi Shodex NH2P‐50 4E column (4.6 × 250 mm). The column temperature was maintained at 50°C. The mobile phase consisted of acetonitrile containing 2% acetic acid (phase A) and an aqueous solution containing 5% acetic acid and 3% triethylamine (phase B). The flow rate was set to 1 mL/min. After sample injection, the concentration of mobile phase B was held at 30% for 2 min, followed by a linear gradient increase to 95% over 80 min. The injected amount of 2AA‐labeled N‐glycans corresponded to 0.3 μL of serum.

**FIGURE 1:**
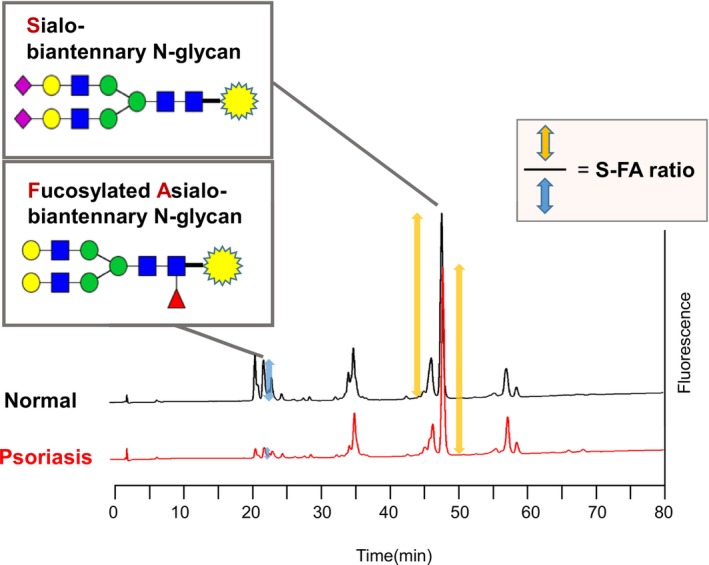
Setting of the S/FA ratio. In a preliminary experiment, the serum level of FA, as determined by high‐performance liquid chromatography, was relatively lower in patients with psoriasis than in healthy controls; in contrast, the S remained nearly constant across all groups. Since these two N‐glycan forms are interrelated through terminal sialylation, we defined the S/FA ratio as a representative glycomic parameter for subsequent analyses. Symbol key for N‐glycan structures: Blue squares, N‐acetylglucosamine; green circles, mannose; yellow circles, galactose; magenta diamonds, sialic acid; red triangles, fucose; yellow star, protein backbone to which the N‐glycan is attached. S, sialylated biantennary N‐glycan; FA, fucosylated asialo‐biantennary N‐glycan; S/FA ratio, ratio of sialylated to fucosylated asialo‐biantennary N‐glycans.

In an exploratory analysis using a small independent set of commercially available serum samples, serum levels of fucosylated asialo‐biantennary N‐glycan (FA) tended to be lower in inflammatory diseases, including psoriasis. In contrast, the sialylated form (S) remained relatively constant across disease groups (not included in this report). Since these two glycan forms represent closely related glycan forms and the use of a ratio can reduce inter‐individual and technical variability, we focused on the ratio of S to FA (S/FA ratio) for subsequent analyses, which were assessed in a blinded manner (Figure 1).

To assess analytical reproducibility, inter‐assay variability of the S/FA ratio was assessed using serum samples from three healthy individuals (Table S1). Measurements were independently performed at two separate analytical facilities under different experimental conditions, including different operators, instruments, measurement periods, and processing timing. The inter‐assay coefficient of variation (CV) for the S/FA ratio was 8.8%, indicating good reproducibility of the glycomic HPLC assay.

### 2.4 Measurement of Serum CCL20 and CRP Levels

Serum CCL20 levels were measured using a commercial enzyme‐linked immunosorbent assay kit (Proteintech Group Inc., Rosemont, IL, USA). Serum CRP levels were determined using a latex agglutination turbidimetric assay (SRL Inc., Tokyo, Japan).

### 2.5 Cases and Serum Samples

This study was approved by the Ethics Committee of the University of Fukui Hospital (No. 0170060). All serum samples used in the study were obtained and stored during clinically required blood draws. Participants were provided with information about the study and given the opportunity to opt out in accordance with the Declaration of Helsinki.

For an exploratory pilot analysis, we used a small independent cohort of patients with major inflammatory dermatoses, including atopic dermatitis (AD, *n* = 8), psoriasis (*n* = 10), autoimmune blistering diseases (*n* = 9), systemic lupus erythematosus (*n* = 5), and systemic sclerosis (*n* = 9). These serum samples were obtained from the main institutional cohort without overlapping with the main analysis.

Comparative analyses were conducted among three groups: 45 patients with psoriasis (age range: 45–64 years; median: 52 years), 12 patients with AD serving as disease controls (age range: 31–52 years; median: 43.5 years), and 19 healthy controls (age range: 35–55 years; median: 45 years). The psoriasis group included 30 patients with psoriasis vulgaris and 15 with psoriatic arthritis; patients with pustular psoriasis and erythrodermic psoriasis were excluded. The PASI was assessed periodically in the psoriasis group.

### 2.6 Statistical Analysis

Statistical analyses were performed using EZR version 1.54. Data are presented as median [range]. The Kruskal–Wallis test was used to compare values among groups, followed by the Steel–Dwass test for pairwise comparisons. Spearman's rank correlation coefficient was used to evaluate associations between variables. Receiver operating characteristic (ROC) curves were constructed to assess the performance of each biomarker, and the area under the curve (AUC) was calculated to quantify performance. A *p*‐value < 0.05 was considered statistically significant.

## 3 Results

This study consisted of three different analyses: (i) an exploratory analysis using an independent cohort of serum samples from patients with various inflammatory diseases, (ii) the main analysis using an institutional cohort comprising patients with psoriasis, AD, and healthy controls, and (iii) longitudinal analyses in a subset of psoriasis patients with paired serum samples obtained before and after treatment.

### 3.1 Serum S/FA Ratio in Skin and Rheumatic Diseases

As a preliminary experiment, serum S/FA ratios were compared among various skin and rheumatic diseases with a small number of cases per group (Figure S1). These diseases include AD, psoriasis, autoimmune bullous diseases (bullous pemphigoid and pemphigus vulgaris), systemic lupus erythematosus, and systemic sclerosis. Although interindividual variation was observed within each disease group, the S/FA ratio in inflammatory skin diseases—such as AD, psoriasis vulgaris, and autoimmune bullous diseases—tended to be higher than in healthy controls, with a particularly marked increase in psoriasis.

Based on these findings, further analyses focused on psoriasis, using AD as a disease control. The clinical characteristics of 45 patients with psoriasis are summarized in Table 1. The median PASI score was 8.9 [0–30.6], and 15 patients (33.3%) had arthralgia. At enrollment, treatments in the psoriasis cohort included biologics (*n* = 21, 46.7%), etretinate (*n* = 7, 15.6%), apremilast (*n* = 5, 11.1%), cyclosporine (*n* = 3, 6.7%), methotrexate (*n* = 2, 4.4%), and ultraviolet therapy (*n* = 2, 4.4%), some of whom received combination regimens of these treatments. 14 patients (31.1%) were not treated with such systemic therapy at the time of serum sampling.

**TABLE 1:** Clinical characteristics of patients with psoriasis.

	Psoriasis (*n* = 45)
Age (median [range])	52.0 [23–81]
Male/Female	36:9 (80.0% male)
PASI (median [range])	8.9 [0–30.6]
Arthralgia	15 (33.3%)
Treatment	
Biologics	21 (46.7%)
IL‐23 inhibitors	8 (17.8%)
TNF inhibitors	7 (15.6%)
IL‐17 inhibitors	6 (13.3%)
Etretinate	7 (15.6%)
Apremilast	5 (11.1%)
Cyclosporine	3 (6.7%)
Methotrexate	2 (4.4%)
Ultraviolet therapy	2 (4.4%)
Comorbidities	
Dyslipidemia	12 (26.7%)
Diabetes	9 (20.0%)
Hypertension	7 (15.6%)
Smoking	17 (37.8%)

*Note:* Data are presented as *n* (%) unless otherwise specified.

Abbreviation: PASI, Psoriasis Area and Severity Index.

The AD cohort had a median EASI score of 17.7 (range 1.4–42.7). One patient received biologics (IL‐4/13 inhibitors), whereas the remaining 11 patients (91.7%) had not received systemic immunosuppressants, biologics, or JAK inhibitors.

### 3.2 Serum S/FA Ratio Is Elevated in Psoriasis

**FIGURE 2:**
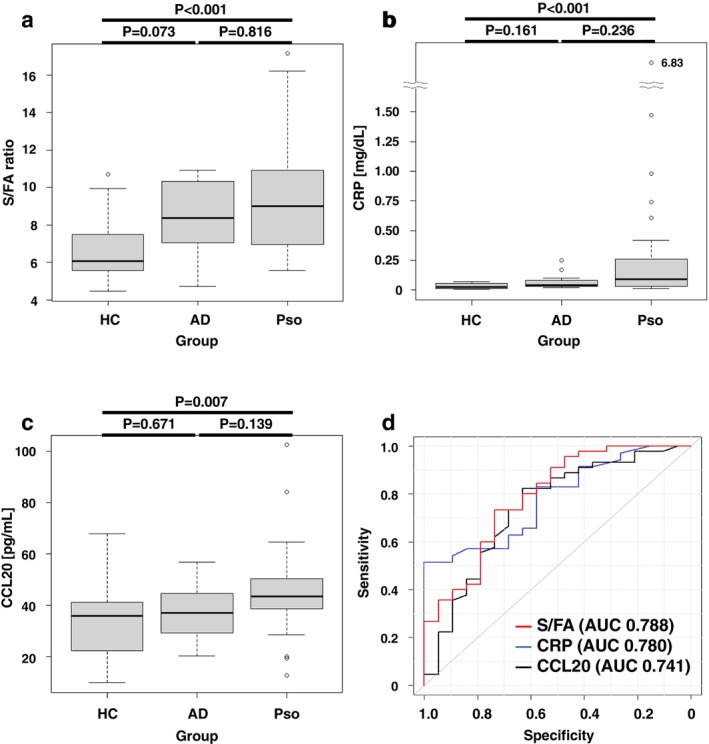
Serum levels of S/FA, CRP, and CCL20 in patients with AD and Psoriasis at baseline. (a) S/FA, (b) CRP, (c) CCL20. Data are presented as box‐and‐whisker plots, showing the median, interquartile range, and potential outliers. (d) Receiver operating characteristic curves showing the AUCs for S/FA, CRP, and CCL20 comparing patients with psoriasis to healthy controls. S/FA, ratio of sialylated to fucosylated asialo‐biantennary N‐glycans; CRP, C‐reactive protein; AD, atopic dermatitis; Pso, psoriasis; AUC, areas under the curve; CCL20, C‐C motif chemokine ligand 20.

We next investigated the serum S/FA ratio in patients with psoriasis and compared it with AD and healthy controls (Figure 2a). Patients with AD showed higher S/FA ratios than healthy controls; however, the difference was not statistically significant (*p* = 0.073). On the other hand, patients with psoriasis exhibited significantly elevated S/FA ratios compared with healthy controls (*p* < 0.001). No significant difference was observed between the psoriasis and AD groups (*p* = 0.816).

### 3.3 Performance of the Serum S/FA Ratio

The performance of the S/FA ratio was compared with that of previously reported psoriasis biomarkers, including CRP and CCL20. Serum CRP levels were significantly elevated in patients with psoriasis compared with those in healthy controls (*p* < 0.001, Figure 2b). In contrast, CRP levels in patients with AD were comparable to those in healthy controls.

Similarly, serum CCL20 levels were significantly higher in patients with psoriasis than in healthy controls (*p* = 0.0070, Figure 2c) but not in patients with AD.

For distinguishing psoriasis from healthy controls, the S/FA ratio showed a sensitivity of 73.7% and a specificity of 73.3% at a cutoff value of 7.207. ROC curve analysis indicated an AUC of 0.788 (95% CI: 0.661–0.916), indicating good performance (Figure 2d). In comparison, CRP showed an AUC of 0.780 (95% CI: 0.657–0.902), with 100% sensitivity and 51.4% specificity at a cutoff of 0.080 mg/dL. In contrast, CCL20 had an AUC of 0.741 (95% CI: 0.598–0.884), with 63.2% sensitivity and 82.2% specificity at a cutoff of 36.762 pg/mL. Thus, the performance of the serum S/FA ratio was comparable to that of CRP and CCL20.

### 3.4 Association Between Serum S/FA Ratio and Clinical Characteristics in Psoriasis

The S/FA ratio showed greater variability in the psoriasis group than in healthy controls. Therefore, a cutoff value of 10.38—defined as the 75th percentile plus 1.5 times the interquartile range (which approximates the 95th percentile of healthy controls)—was used to classify patients with psoriasis into S/FA ratio–elevated and non‐elevated subgroups.

Comparison of clinical features between these two subgroups revealed associations with PASI and age. Patients in the S/FA ratio‐elevated subgroup had significantly higher PASI scores (8.8 vs. 1.6, *p* = 0.006) and were younger (46.5 vs. 54.0 years, *p* = 0.028) than those in the non‐elevated subgroup (Table 2). However, no correlation between S/FA ratio and age was found in healthy individuals or in patients with AD, suggesting that the inverse correlation in psoriasis may be driven by a higher proportion of severe cases among younger patients. Other factors, including sex, arthralgia, hypertension, diabetes, dyslipidemia, and smoking, did not differ significantly between subgroups.

**TABLE 2:** Association between the S/FA ratio and clinical characteristics.

	S/FA ratio non‐elevated (*n* = 30)	S/FA ratio elevated (*n* = 15)	*p*
Male/Female	23:7 (76.6% male)	13:2 (86.7% male)	0.695^a^
Age (median [range])	54.0 [23–81]	46.5 [27–66]	0.028^b^
PASI (median [range])	1.6 [0–19.9]	8.8 [0–30.6]	0.006^b^
Arthralgia	11 (36.7%)	4 (26.7%)	0.738^a^
Hypertension	5 (16.7%)	2 (13.3%)	1.000^a^
Diabetes	6 (20.0%)	3 (20.0%)	1.000^a^
Dyslipidemia	7 (23.3%)	5 (33.3%)	0.496^a^
Smoking	9 (30.0%)	8 (53.3%)	0.193^a^

*Note:* Data are presented as *n* (%) unless otherwise specified.

Abbreviation: PASI, Psoriasis Area and Severity Index.

^a^ Chi‐square test.

^b^ Mann–Whitney *U* test.

### 3.5 Association of Serum S/FA Ratio With Disease Severity

**FIGURE 3:**
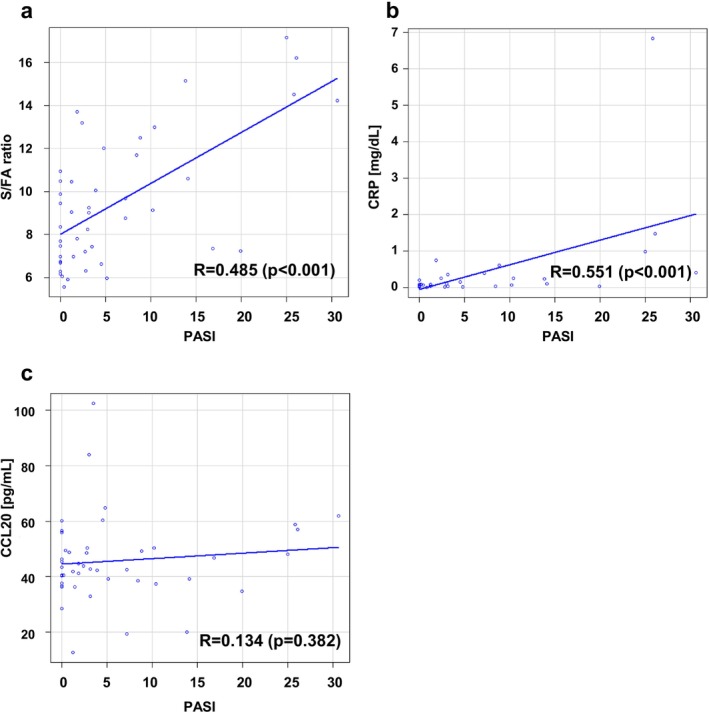
Correlation between baseline serum levels of S/FA, CRP, and CCL20 and PASI in patients with psoriasis. Scatter plot showing the correlation between PASI and S/FA ratio at baseline. Each dot represents an individual patient. The blue line indicates a fitted linear regression line for visualization. S/FA, sialylated to fucosylated asialo forms; CRP, C‐reactive protein; PASI, Psoriasis Area and Severity Index; CCL20, C‐C motif chemokine ligand 20.

The serum S/FA ratio was significantly correlated with PASI in patients with psoriasis (*r* = 0.485, *p* < 0.001; Figure 3a). Similarly, serum CRP levels correlated with PASI (*r* = 0.551, *p* < 0.001; Figure 3b), but CCL20 levels showed no significant correlation (*r* = 0.134, *p* = 0.382; Figure 3c). These findings suggest that the serum S/FA ratio is associated with psoriasis severity, similar to CRP.

Further analyses assessed the relationship between the S/FA ratio and three individual PASI components. The S/FA ratio showed significant positive correlations with erythema (*r* = 0.444, *p* < 0.01), induration (*r* = 0.425, *p* < 0.01), and scaling (*r* = 0.432, *p* < 0.01), with no clear differences in correlation strength among these components (Figure S2a–c, respectively). In addition, the S/FA ratio was significantly correlated with Body Surface Area (BSA, *r* = 0.409, *p* = 0.01) (Figure S2d).

Across all biomarkers, no consistent clinical characteristics were observed among cases with outlier values. The patient with a CRP level of approximately 7 had PsA and showed poor control of both cutaneous and joint symptoms, possibly related to suboptimal adherence to TNF inhibitor therapy.

When restricted to patients with psoriasis vulgaris (*n* = 30), excluding PsA (*n* = 15), the correlation between the S/FA ratio and CRP levels was almost comparable (*r* = 0.573, *p* < 0.001 and *r* = 0.575, *p* < 0.001, respectively) (Figure S3). In contrast, serum CCL20 levels showed no correlation with PASI (*r* = 0.234, *p* = 0.71). These findings indicate that the S/FA ratio may be more closely associated with cutaneous disease severity than with arthralgia.

### 3.6 Association of Serum S/FA Ratio With Treatment Response

Paired serum samples were available for 43 patients with psoriasis before and after treatment, allowing assessment of biomarker changes. Treatment regimens included IL‐17 inhibitors (*n* = 14), IL‐23 inhibitors (*n* = 14), TNF inhibitors (*n* = 12), cyclosporine (*n* = 2), and apremilast (*n* = 1). The median duration was 91 days (range, 28–1799 days).

**FIGURE 4:**
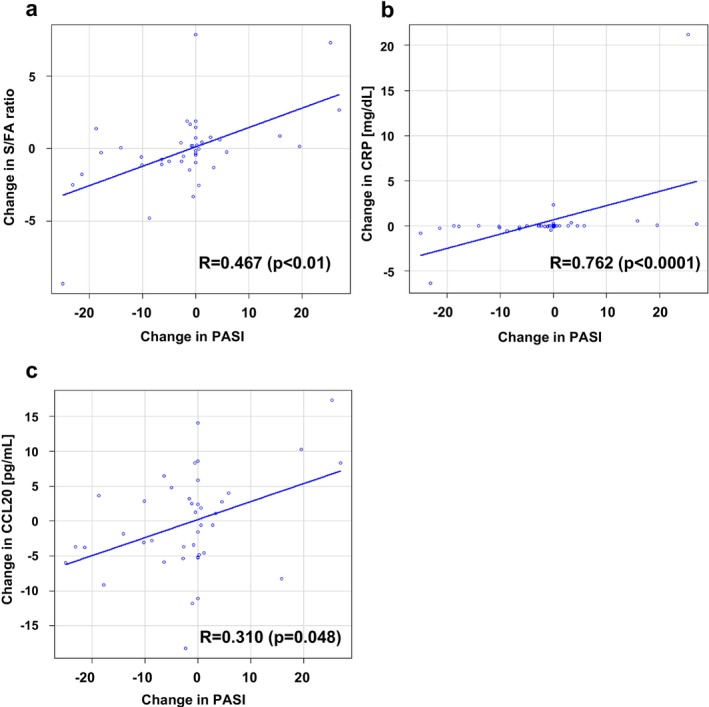
Correlation between changes in serum levels of S/FA, CRP, and CCL20 and changes in PASI before and after each biologic therapy in patients with psoriasis. Scatter plot showing the correlation between the change in the PASI score and the S/FA ratio before and after treatment. Each dot represents an individual patient. The blue line indicates a fitted linear regression line for visualization. S/FA, ratio of sialylated to fucosylated asialo‐biantennary N‐glycans; CRP, C‐reactive protein; PASI, Psoriasis Area and Severity Index; CCL20, C‐C motif chemokine ligand 20.

Changes in the serum S/FA ratio significantly correlated with improvements in PASI after treatment (*r* = 0.467, *p* < 0.01; Figure 4a). Changes in CRP levels also showed a strong correlation with PASI (*r* = 0.762, *p* < 0.001; Figure 4b). In contrast, changes in serum CCL20 levels demonstrated a moderate correlation (*r* = 0.310, *p* = 0.048; Figure 4c). These results indicate that the S/FA ratio may serve as a biomarker reflecting treatment response in psoriasis, similar to CRP and CCL20.

### 3.7 Differences in the Association Between Changes in the S/FA Ratio and PASI by Biologic Class

We next examined whether the correlation between changes in the S/FA ratio and PASI differed according to the class of biologic agents (Figure S4). Despite the limited number of patients in each subgroup, a significant correlation was observed in the IL‐23 inhibitor‐treated group (*r* = 0.652, *p* = 0.016), whereas no significant correlation was observed in the other two treatment groups (TNF inhibitors, *r* = 0.410, *p* = 0.164; IL‐17 inhibitors, *r* = 0.486, *p* = 0.129).

## 4 Discussion

In this study, we found that the serum S/FA ratio, a glycan‐based biomarker candidate, was significantly elevated in patients with psoriasis compared with healthy controls and was associated with disease severity and treatment response. Notably, its performance, correlation with PASI, and responsiveness to treatment were substantially equivalent to those of previously reported biomarker candidates, such as CRP and CCL20. To our knowledge, this study is among the first to suggest that a serum glycan ratio may serve as a biomarker associated with both disease severity and treatment response in psoriasis.

A previous study reported that serum N‐glycan profiles could serve as diagnostic biomarkers for psoriasis by comparing patients with psoriasis and healthy controls using DNA sequencer‐assisted fluorophore‐assisted carbohydrate electrophoresis [9]. Distinct differences in N‐glycan structures were observed, with four elevated and four decreased peaks in patients with psoriasis compared with controls. In contrast to this approach, our study utilizes a ratio‐based glycomic parameter (S/FA ratio), which may reduce inter‐individual variability and better reflect disease‐associated glycan remodeling.

Regarding the in vivo sources of N‐glycans, FA (fucosylated asialo‐biantennary N‐glycan), which constitutes the denominator of the S/FA ratio, is mainly derived from IgG Fc glycans, a core‐fucosylated biantennary structure, and their glycosylation profiles are known to be altered in chronic inflammatory and autoimmune diseases [7, 10, 11, 12]. More specifically, IL‐23/Th17‐ and TNF‐driven inflammatory pathways can influence these B‐cell glycosylation pathways [11], which may provide a possible explanation for the reduced FA levels in our cohort.

In contrast, sialo‐biantennary N‐glycans (S) represent a composite signal derived from multiple serum glycoproteins, including α2‐HS‐glycoprotein (fetuin‐A) and acute‐phase proteins (e.g., α1‐acid glycoprotein and haptoglobin) [13, 14], as well as other abundant serum glycoproteins such as transferrin, rather than reflecting a single specific carrier protein. Notably, fetuin‐A is a negative acute‐phase protein whose levels decrease under inflammatory conditions [15], whereas several acute‐phase glycoproteins exhibit the opposite behavior. Because glycomics characterizes the overall glycan composition of a given sample rather than glycosylation on specific carrier proteins [16], changes in one glycan source may be offset by contributions from others. In this context, the preferential reduction in FA, together with relatively preserved S levels, may contribute to the observed increase in the S/FA ratio in psoriasis.

Together with these, psoriasis and AD are characterized by distinct immune polarization: a predominant IL‐23/Th17 axis in psoriasis and a Th2‐skewed inflammation in AD. The differential cytokine milieus may influence B‐cell maturation and resultant IgG productivity and glycosyltransferase activity, thereby contributing to disease‐related glycome remodeling. However, the specific carrier proteins for FA and S cannot be identified in the current dataset and need to be investigated in future studies.

The serum S/FA ratio tended to be higher in psoriasis than in AD and other inflammatory diseases; the difference did not reach statistical significance, and no conclusion can be drawn regarding disease specificity. This may be partly explained by differences in patient background, as many AD patients had relatively high disease activity without systemic treatment, whereas most psoriasis patients were already receiving systemic therapies. This imbalance in treatment background between the cohorts likely influenced both the observed S/FA ratio and the comparison between disease groups, substantially limiting the interpretation of disease specificity. Nevertheless, the S/FA ratio showed significant correlations with all three PASI components and remained associated with disease severity in the subgroup with skin disease alone (psoriasis vulgaris), suggesting a close relationship with skin manifestations of psoriasis.

Another interesting finding of this study is the correlation between changes in the S/FA ratio and PASI across different biologic classes. The association appeared stronger in the IL‐23 inhibitor‐treated group, whereas no significant correlation was observed in the IL‐17‐ or TNF inhibitor‐treated groups. These findings suggest a possible link between the S/FA ratio and IL‐23/Th17‐related immune pathways, although this observation should be interpreted with caution given the exploratory nature of the analysis and the limited sample size. Although AD is classically characterized by Th2‐skewed inflammation, involvement of the IL‐23/Th17 pathway has also been reported, particularly in Asian populations [17]. In our Japanese cohort, the S/FA ratio was elevated not only in psoriasis but also in AD, which may reflect the influence of IL‐23/Th17‐related immune pathways on the S/FA ratio. Since IL‐23 acts upstream in maintaining pathogenic Th17 responses, its blockade may lead to a more coordinated normalization of both skin inflammation and glycomic alterations. Future studies are needed to determine whether glycomic changes differ among single cytokine‐targeted therapies under more consistent clinical conditions.

CRP is a general acute‐phase reactant that represents non‐specific, systemic inflammatory signals and has been reported to correlate with disease severity in psoriasis [2, 3]. However, CRP levels may be influenced by extracutaneous inflammatory involvement, including subclinical arthritis or comorbidities, complicating the assessment of skin disease severity. In our cohort, CRP levels tended to remain low in most patients despite variability in PASI scores. In contrast, the S/FA ratio showed a broader distribution with less skewness than CRP and may serve as a complementary biomarker.

CCL20 expression was significantly higher in psoriatic lesional skin than in non‐lesional skin or healthy control skin [18]. CCL20, produced mainly by keratinocytes, recruits T cells—particularly Th17 cells—which in turn secrete IL‐17 and IL‐22, cytokines critical for the development and maintenance of psoriasis [19]. The fact that symptoms had improved with prior systemic therapies such as biologics or immunosuppressants at baseline may partly explain why serum CCL20 levels did not strongly correlate with psoriasis severity in our cohort, in contrast to the previous report [5].

This study has several limitations. Its retrospective, single‐center design and modest sample size may limit the generalizability of the findings. In addition, most patients with psoriasis, but not those with AD, were already receiving systemic treatments at baseline, which may have influenced disease severity, disease‐control comparisons, disease specificity, and biomarker levels. Although significant correlations between the S/FA ratio and PASI were observed, the potential impact of residual confounding factors, including age, sex, and comorbidities, cannot be fully excluded. Inclusion of additional disease controls, such as hidradenitis suppurativa, a disease that partially shares pathogenic TNF and IL‐23/Th17‐mediated pathways with psoriasis, would strengthen the assessment of disease specificity and should be addressed in future studies.

Despite these limitations, the S/FA ratio was consistently associated with disease severity and treatment response in psoriasis, with an exploratory assessment for a relatively stronger correlation in the IL‐23 inhibitor‐treated subgroup among biologic therapies. This biomarker may provide complementary information to conventional inflammatory markers such as CRP, potentially enabling more precise monitoring of skin disease severity. Future prospective and longitudinal studies with larger, treatment‐naïve cohorts are required to validate these findings and to further elucidate the temporal relationship between glycan remodeling and clinical outcomes in psoriasis. In addition, N‐glycan profiles may not only enhance biomarker exploration but also provide insight into as yet unidentified treatment strategies targeting glycosylation pathways to modulate inflammatory responses in psoriasis.

## Funding

This study was supported by Sumitomo Chemical Co. Ltd. (Osaka, Japan); Research Grants from the Japan Foundation for Applied Enzymology (Nishimura K); and Research Grants from the University of Fukui.

## Ethics Statement

This study was approved by the Ethics Committee of the University of Fukui Hospital (No. 0170060). Information disclosure and the opportunity to opt out were provided in accordance with the Declaration of Helsinki.

## Conflicts of Interest

Minoru Hasegawa serves as an Editorial Board member of the Journal of Dermatology. Kiyoshi Higashi and Koichi Saito are employees of Sumitomo Chemical Co. Ltd. This study was partly supported by a commissioned research grant from Sumitomo Chemical Co. Ltd. to the Department of Dermatology, University of Fukui.

## Supporting information


**Figure S1:** Serum S/FA ratios were compared in several skin and/or systemic inflammatory diseases.

**Figure S2:** Serum S/FA ratios correlated with Psoriasis Area and Severity Index (PASI) sub‐scores and Body Surface Area (BSA) in patients with psoriasis.

**Figure S3:** Serum S/FA ratios correlated with PASI in psoriasis vulgaris patients without psoriatic arthritis.

**Figure S4:** Correlation between changes in S/FA ratio and PASI before and after treatment according to biologic classes: IL‐17 inhibitors, IL‐23 inhibitors, and TNF inhibitors.

**Table S1:** Inter‐assay coefficient of variation for the S/FA ratio.

## Data Availability

The data that support the findings of this study are available from the corresponding author upon reasonable request.
