# Supplementary material for: A Serum N‐Glycan Ratio Associated With Disease Severity and Treatment Response in Psoriasis
Source: J Dermatol. 2026 Jun 23;53(8):1090–8. doi: 10.1111/1346-8138.70316 (PMC13435095; doi:10.1111/1346-8138.70316)
Supplement: Supplementary file 1 [file JDE-53-1090-s005.docx]

**A Serum N-glycan Ratio Associated with Disease Severity and Treatment Response in Psoriasis**
Kentarou Nishimura, Noritaka Oyama, Takumi Hasegawa, Hiroshi Kasamatsu, Takenao Chino, Takahiro Tokunaga, Kiyoshi Higashi, Koichi Saito, Keita Yamada, Minoru Hasegawa

**Table S1. Inter-assay coefficient of variation for the S/FA ratio.**

| **S/FA ratio** | **Sample1** | **Sample2** | **Sample3** | **Mean** |
| --- | --- | --- | --- | --- |
| **Facility A** | 25.13 | 24.82 | 31.41 | 27.12 |
| **Facility B** | 26.79 | 26.62 | 37.53 | 30.31 |
| **Difference (A-B)** | -1.66 | -1.8 | -6.12 | -3.19 |
| **SD** | 1.17 | 1.27 | 4.33 | 2.54 |
| **Overall mean (all samples)** | 28.72 | | | |
| **Inter-assay CV (%)** | 8.83 | | | |

SD, standard deviation; CV, coefficient of variation.
